# A Policy and Regulatory Framework to Promote Care Delivery Redesign and Production Efficiency in Health Care Markets

**DOI:** 10.1111/1468-0009.70016

**Published:** 2025-05-06

**Authors:** DENNIS P. SCANLON, JILLIAN B. HARVEY, CHERYL L. DAMBERG, PRATIKSHA MAHENDRA BHAGAT, YUNFENG SHI

**Affiliations:** ^1^ The Pennsylvania State University; ^2^ The Medical University of South Carolina; ^3^ RAND Corporation

**Keywords:** health systems, care delivery redesign, consolidation, innovation, efficiency, antitrust enforcement, health care prices, health care costs

## Abstract

**Policy Points**

- Antitrust enforcement has been too narrowly focused on predicting postmerger market share and not enough on the likely impact of mergers and acquisitions on production efficiency and quality.
- Care delivery redesign is a term that captures various innovations and changes in the organization and delivery of health care, which may lead to increased production efficiency and improved quality of care. Regulators and policymakers can use the framework to develop empirical measures to assist in understanding changes in production processes as well as in resultant outcomes.
- Significant opportunities exist to improve data collection and require reporting to better assist regulators with antitrust enforcement and help policymakers create effective legislation. Examples include improving compliance with required hospital and insurer transaction price data reporting, growing the availability of all‐payer claims databases, improving existing Medicare cost reporting, and achieving consensus on quality measures that are best used to measure the impact of consolidation.
- There is a fundamental need to systematically track health care organizations and their affiliations and component parts (e.g., hospitals, physician practices, skilled nursing facilities, etc.) longitudinally, especially as organizations expand across markets and state boundaries and are owned by various entities, including private equity.

Policymakers and the media have been increasingly attentive to mergers and acquisitions and other potentially anticompetitive practices of hospitals, physicians, and other health care providers. Consolidation has the potential to increase efficiency and help some struggling providers keep their doors open in relatively underserved areas. Still, it can also reduce market competition and ease pressure on providers to lower prices or invest in quality improvements. A substantial body of evidence shows that consolidation has led to higher prices without clear evidence of improvements in quality, which has implications for consumers and employers.^1^

This excerpt from a recent kaiser family foundation research brief summarizes the typical story line and concern with any proposed health care merger or acquisition. Such concern is now unsurprisingly widespread, given the dramatic trend of consolidation in health care over the past three decades in the United States, resulting in highly concentrated markets with large health systems currently controlling a significant proportion of care delivery across the country.^2^, ^3^ The formation and growth of health care systems can occur through the horizontal combination of like providers (e.g., hospitals) or the vertical integration of different levels of providers either through acquisition or affiliation, such as primary care or single or multispecialty physician practices in addition to hospitals.^4^ Health systems may also operate and acquire in the insurance or risk industry, adding another dimension to an already complex and multidimensional organizational structure. Although the specific markets as well as the acquiring and acquired contestants may vary, the purported claims regarding the likely benefits of consolidation made by those who seek to combine do not change nor do the concerns raised about the potential negative consequences likely to be experienced by consumers, practicing providers such as physicians, and taxpayers that fund public health benefit programs. For example, proposed mergers and acquisitions are typically justified on the promise of production efficiencies, quality improvements through improved care coordination, and better patient/consumer experiences. Regulators and consumer watchdogs, on the other hand, worry about increased leverage to raise transaction prices by the consolidated entity, even if the newly formed organization may experience operational efficiencies. However, even with aggressive and successful antitrust regulation and enforcement, which are mainly designed to prevent further consolidation, it may be practically challenging to increase competition and reduce concentration from the status quo.^5^ In such cases where markets are already highly concentrated, the regulatory and policy goal should be to ensure that access and quality do not suffer and that prices are fair. Importantly, not all consolidation in health care is necessarily negative and can indeed be beneficial to consumers and society, but the potential positive side is unlikely to materialize without policy and regulatory oversight.^2^, ^6^ To assess the consequent harms and benefits of consolidation in specific and tangible ways, it is crucial to understand how the various aspects of restructuring, reorganization, and potentially innovative redesign in how care is delivered may improve quality and efficiency and reduce cost.^7^, ^8^

In this article, we discuss why reliance on transaction prices and market share alone is not sufficient for effective health policy development and regulatory enforcement in health care markets that are imperfectly competitive. We discuss the need to better measure the output produced by health care suppliers and to capture the costs of producing that output. We also discuss the need to measure transaction prices more accurately and to tie these prices to the cost of production. For illustrative purposes, we focus on one health care provider type that has grown significantly in recent years, often through consolidation that promises better outcomes at lower prices: health care delivery systems. We discuss health systems and their specific production objective, termed care delivery redesign (CDR), with the goal of measuring and monitoring the implementation and impact of CDR to understand how it is related to transaction prices, costs of production, and quality of output produced.

A better understanding of how care is produced and if it is being produced efficiently can be useful from both a regulatory and a policy perspective. From a regulatory perspective, proposals for mergers or acquisitions often pose challenges to the Federal Trade Commission (FTC), the Department of Justice (DOJ), and state‐level authorities who need to assess whether the potential harm due to increased market power should warrant approval or if to impose an injunction or other interventional remedy under antitrust laws to protect consumers.^9^, ^10^, ^11^ Ideally, such assessment, inherently predictive in nature, should include the impact of proposed consolidation on production and operational efficiency, transaction prices, and output quality because entities proposing consolidation often promise improvements in these areas, which can be difficult to measure and monitor. Regulators, thus, need help to more concretely understand the feasibility of the proposed benefits, which can be weighed against the possible harms. Moreover, when certain consolidations may be approved but require various remedies, such as price restrictions or service commitments,^5^ regulatory policies will need to set appropriate and reasonable conditions on which the consolidations are contingent and monitor these conditions prospectively. For example, independent rural hospitals are often in financial distress and at risk of closure, which can lead to significantly reduced care access for their local communities.^12^, ^13^ Becoming absorbed by a large health system may be the only practical option for some hospitals to survive.^14^ From a policy perspective, because there are many areas of the country where health care is already highly concentrated or even provided by a monopoly and the influx of additional competitors is unlikely, effective federal and state health policies and well‐designed payment structures are critical to incentivizing efficiency and quality in care delivery and to abate the potential negative effects of highly concentrated markets. Although it may be impractical to maintain full access to every service across all geographic areas at the highest level of quality, efficient production should always be the goal conditional on any thresholds of access and quality that can be realistically established for a specific region.

Beyond antitrust regulation, something more is clearly needed to achieve the outcomes desired by consumers, payers, and policymakers, as spending on health care in the United States, largely driven by price increases, has been consistently higher than other peer nations by far and has generated quality of care and health outcomes that are generally worse.^15^, ^16^, ^17^ In addition, price and quality of care within the United States vary significantly across different regions and providers for reasons that cannot be explained by patient case mix or cost of living differences.^18^, ^19^ Even though there has been sufficient evidence suggesting that market consolidation leads to increased prices,^20^, ^21^ it is not clear to what extent the prices we pay for care reflect the actual value, quality, and costs of production.^22^, ^23^ Health care in the United States is known for being inefficient and wasteful,^24^, ^25^, ^26^ not in small part owing to limited price and quality transparency as well as misaligned incentives across providers, patients, and payers (i.e., what has been described as the “agency problem”). These issues collectively make it a serious challenge to effectively improve quality while reducing (or at least controlling) spending as epitomized in the “Quadruple Aim” framework (formerly “Triple Aim”).^27^, ^28^ At the core of this challenge is the need for better measurement of quality and efficiency in health care production and delivery and their links to prices under different market conditions. Despite all the efforts on price and quality transparency in health care over the past several decades, our ability as a society to monitor price and quality and to inform decisions by consumers, payers, and policymakers based on them is still immensely limited.^29^ Moreover, there are concerns that the measures currently used in investigating health care consolidation may have missed important aspects of changes in care delivery.^30^, ^31^

The term CDR refers to various forms of innovations and changes in delivering care with the aim(s) of improving production/operational efficiency and/or care quality.^8^ Most health care providers and health systems have been engaged in some CDR efforts, partly driven by payment incentives and regulatory policy initiatives from the public and the private sectors and motivated by the persistently high cost and low‐quality health care.^8^, ^32^, ^33^, ^34^ However, the level of engagement in CDR varies significantly across providers and has not been formally operationalized in policy and regulatory decision making.^8^

## Transaction Price Data Are Not Sufficient to Regulate Imperfectly Competitive Health Care Markets: the Need for Production Function and Output Data

The theory of the firm, as described in microeconomics, is helpful for thinking about the challenges and limitations of regulating health care markets and developing effective health policies to address potential market failures. That theory states that in perfectly competitive markets, where the product being produced and sold is homogenous (e.g., many agricultural products such as corn or soybeans), there are many producers/suppliers. There are few, if any, barriers to entry, so price should converge to the efficient cost of production because inefficient producers will fail. In this market structure, price alone should be sufficient to signal production efficiency and product quality. However, many markets for goods and services are not perfectly competitive owing to real or perceived product and service differentiation (i.e., nonhomogenous output), limited numbers of suppliers, nontransparent information about product/service price and quality, and barriers in the ability to enter and compete in the marketplace, including in some cases significantly prohibitive fixed investment costs to operate. Although some health care markets may meet the conditions of perfect competition (e.g., markets for the collection and processing of standard laboratory tests), most do not, which limits the ability to rely on transaction prices alone for purposes of assessing production efficiency and value for consumers and society. As one moves beyond perfect competition to other market structures—what microeconomics describes as monopolistic competition, oligopoly, or monopoly market structures—it is essential to not just measure transaction prices but to measure the cost of production (i.e., production efficiency) and the quality of the output, which by default is heterogenous (i.e., different in reality and/or the perception of consumers). This is a critical point because so much attention has been placed on measuring health care transaction prices in the United States, an endeavor that is useful but has been highly criticized as ineffective for reasons discussed in the section “Enforce 2019 Executive Order on Improving Health Care Price and Quality Transparency,” while insufficient effort has been placed on measuring output heterogeneity and production efficiency, constructs that are critical to measure in addition to price if one is to successfully regulate imperfect markets. It would be unfair to say that there has been no attention paid whatsoever to the production of health care services. For example, Medicare has required the reporting of its health care cost reports by hospitals for years,^35^ and some of Centers for Medicare and Medicaid Service's (CMS) recent payment methods have tried to incentivize efficiently produced “care bundles” through the Bundled Payments for Care Improvement (BPCI) initiative.^36^ Over the past four decades, there has been significant growth in the health care quality measurement industry, which has been soundly criticized for having produced too many measures that are both burdensome to collect but also challenging to use for their intended purposes and may even drive up health care expenditures and result in suboptimal care quality and outcomes, a concern raised by Blumenthal and McGinnis:

Not only are many [quality] measures imperfect, but they are proliferating at an astonishing rate, increasing the burden and blurring the ability to focus on issues most important to better health and healthcare. Measures of the same phenomenon also vary in specification and application, leading to confusion and inefficiency that make healthcare more expensive and undermine the very purpose of measurement, namely, to facilitate improvement.^37^

It would not be unreasonable to conclude that health policy and regulation has largely been unsuccessful based on the inability to control significant cost growth and to ensure fair pricing for efficiently produced health care transactions that provide socially desirable access and care quality. For this to change, the United States needs to improve available transaction price and quality data and begin to measure in earnest both the process and costs of health care production. Accomplishing this objective will require both rethinking how to measure these important constructs and a commitment to collecting and analyzing the necessary data for purposes of informing health policy and regulation. In the next section, we discuss an approach for measuring and assessing production efficiency using the health care system as the example of the producer. Consistent with the Agency for Healthcare Research and Quality's (AHRQ) Comparative Health Systems Performance initiative,^4^, ^7^, ^8^, ^31^, ^38^ health systems are defined as organizations that include “at least one hospital and at least one group of physicians that provide comprehensive care”.^39^, ^40^ We end by discussing practical suggestions for how existing efforts to measure transaction prices and care quality can be improved and linked to information about care production to ultimately benefit better policy formation and more effective regulation.

## Health Care System Production: the Concept of CDR

To illustrate the importance of understanding and measuring what health care firms produce, both in terms of output and cost efficiency, we focus our example on organized health care delivery systems because they are a major force driving consolidation in health care markets, with many health systems now commanding resources much greater than what was traditionally available to individual hospitals, physician practices, or other types of care delivering entities (e.g., stand‐alone long‐term care facilities).^41^ With these expanded resources, health systems could plausibly have the capacity to implement large‐scale changes that could result in improved production and operation efficiencies and improvements in quality, even if increased market concentration and reduced competition can also provide leverage to increase prices and/or lower the quality of care for consumers.^42^, ^43^, ^44^, ^45^ Therefore, understanding the role of health systems in care delivery transformation has important implications for market‐based policymaking.^46^

Our framework is drawn from prior work on this topic and incorporates multilayered contextual factors affecting CDR implementation and the links between CDR interventions and key outcomes, including changes in the organizational structure, production/operations, and quality of care. To put it into economic terms, the focus of CDR is to understand how health care systems turn raw inputs—such as labor, capital, care innovations, management techniques, technology, and other resources—into what they produce: ideally patient access to timely, effective, and equitable health care services and treatments at sufficient levels of quality that provide patients with a satisfying experience. This is, of course, a simplified description because health care systems are large entities with multiple providers, facilities, and patients and provide many types of care (e.g., diagnostic, curative, preventive) for many different clinical conditions (e.g., diabetes, heart disease, wellness visits) and for many different patient populations (e.g., children, those with chronic illness, disabled, the elderly). Unlike many industries in which standard production technology is easily automated, replicated in volume, and controlled with a high degree of certainty (e.g., Six Sigma), health care delivery is highly dependent on human interactions and behavior and is subject to significant clinical uncertainty. Health care systems may best be viewed as multiproduct firms, making it complex to easily capture, let alone measure, what they produce (e.g., outpatient visits, hospital discharges, care episodes, quality adjusted life years, etc.). They potentially require an approach that focuses on a system's component parts (e.g., hospitals and ambulatory practice sites) while somehow aggregating to the system level. Nonetheless, because health systems negotiate prices with payers, and fair transaction prices should be linked to efficient production, it is critical that health policy and health regulation do a better job of measuring health care production.

## Existing Literature on CDR

A significant body of research has been developed in the areas of care transformation and care delivery improvement,^47^ with most studies focusing on one specific type of redesign intervention rather than the wholesale transformation of a provider's health care production function. The diverse topics of these studies reflect the broad scope of CDR, ranging from interprofessional team‐based approaches^1^ (i.e., forming integrated care teams including members from different professions) to the adoption of various electronic health record (EHR) functionalities,^48^, ^49^ data‐driven practice‐based population management approaches, and the use of wireless technology in enhancing patient‐provider communication.^50^ The literature points to four primary factors driving the focus on CDR: (1) the reallocation of risks and performance‐based incentives in various new payment models; (2) the formation of new provider organizations such as accountable care organizations, integrated delivery systems, and clinically integrated networks^51^, ^52^, ^53^; (3) the advancement of health information technology (HIT); and (4) the deployment of various care management approaches and innovative care models designed to improve process efficiency and/or quality of care.^39^

There are three important takeaways from this literature. First, CDR is often used interchangeably with other related terms to describe this latent construct, including “care transformation,” “reengineering care,” “patient‐centered care,” and “value‐based care.” Many of these terminologies have evolved from various approaches used in quality improvement. Second, despite the trend of market consolidation and the growth of health systems, the published research about CDR in health systems is seldom focused on the overarching strategic production goals of the system, with most articles focusing singularly and independently on specific care transformation interventions planned or implemented by a system's individual component entities. For example, certain hospitals or physician practices sometimes only briefly mention health systems in the background without describing how the specific change fits into the overall organizational strategy.^50^, ^54^, ^55^, ^56^, ^57^, ^58^, ^59^ Third, a large number of studies describe process improvement or care delivery changes across very different health systems and care settings (e.g., primary care, specialty care, emergency care), different patient populations (e.g., geriatric care, pediatric care), and different clinical areas (e.g., diabetes care, oncology care).^60^, ^61^, ^62^, ^63^, ^64^, ^65^, ^66^, ^67^, ^68^, ^69^, ^70^, ^71^, ^72^, ^73^ In sum, there is a lack of coherent conceptualization of CDR that connects these varied approaches and interventions to changes in the outcomes that matter: more efficient production, improved quality, and better care access and patient experience.

## A Conceptual Framework for CDR in Health Systems

A valuable conceptual framework has at least two purposes: (1) to formally acknowledge that health systems are responsible for transforming raw inputs into producing service outputs at specific levels of care quality and production efficiency and to connect these outputs to observed transaction prices, necessitated by the fact that most health care markets are not perfectly competitive as discussed previously; and (2) for highlighting the critical need for better data and measurement tied to the conceptual framework to support more effective policy formation and regulatory oversight.

**Figure 1:**
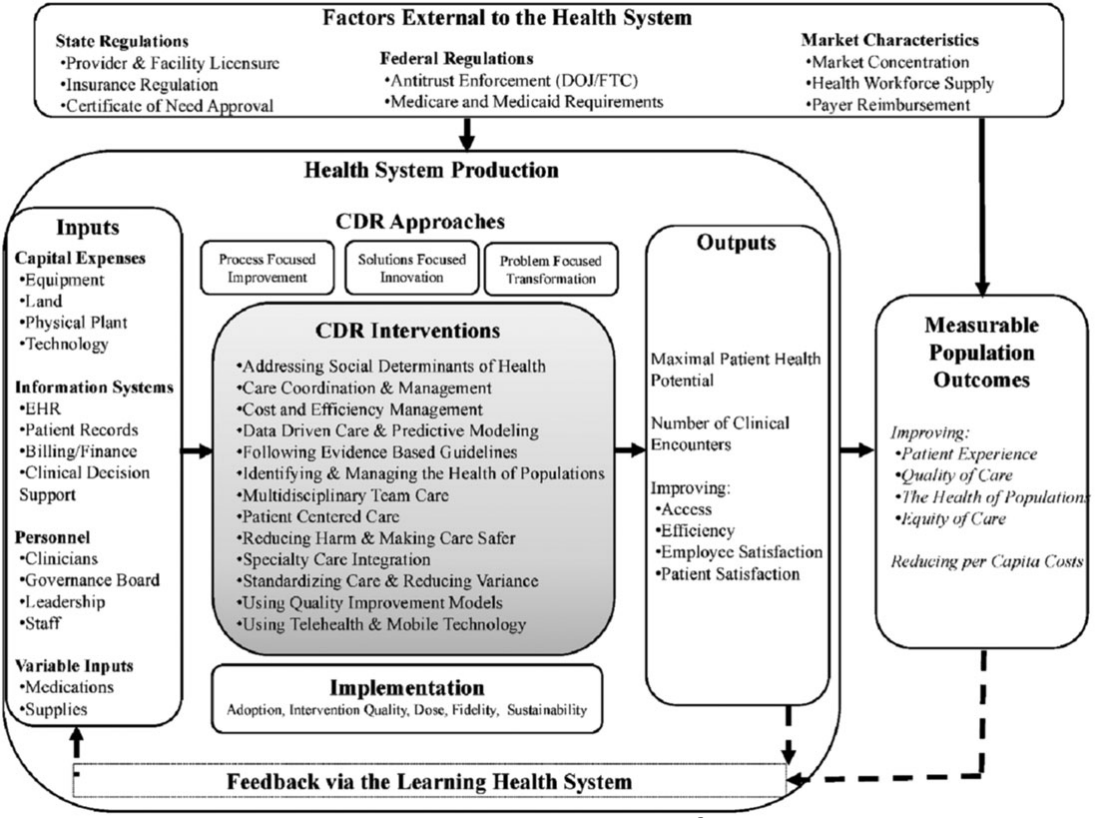
A Conceptual Model for Producing Innovations in Care Delivery Redesign. CDR, care delivery redesign; DOJ, Department of Justice; EHR, electronic health record; FTC, Federal Trade Commission.

As shown in the far‐right side of the framework in Figure 1, health systems’ CDR efforts are intended to impact production outputs: the quality and cost of what is produced and delivered, in aggregate commonly referred to as the “Quadruple Aim.”^27^, ^74^ These Quadruple Aim outcomes reflect the care provided across a health system's attributed patients and are consistent with the primary areas of improvement typically promised when entities seek regulatory approval for growth through consolidation: improvements in population health (e.g., improved health status, reduced disease burden), improved patient experience and care quality (e.g., better satisfaction, care that is safe, timely, effective, equitable, efficient, and patient‐centered care), and reduced per capita cost of care.^74^, ^75^ Importantly, although health systems promise significant improvement in these dimensions, improvement is not guaranteed and will only materialize with time if health systems can successfully implement and manage a combination of strategic production innovations, dedicated resource investment, and formal process improvements. Often these changes involve substituting one type of input for another (e.g., using information technology [IT] in place of some tasks performed by humans) or organizing complementary inputs to work more productively and efficiently together (e.g., team‐based care models that bring together health care workers with complementary skill sets).

## Figure 1: Framework for CDR

As one moves to the left in the Figure, it should be clear that the desired outcomes, or those proposed by potential consolidators, require myriad changes related to a health system's internal infrastructure as well as specific process enhancements and associated interventions (i.e., the middle portion of the framework labeled “CDR approaches”). The far left of the Figure highlights factors that are more or less mutable such as physical plant capacity, organizational governance, and provider workforce composition for example. Of course, the ability to change many of these factors is what presumably motivates the desire for organizations to merge and acquire. Consolidation alone, or what some call “bigger is better,” does not necessarily ensure better outcomes. Critical to scalable and sustainable CDR is the need for buy‐in from individual departments or providers that will champion the CDR work and clear strategic and operational direction from management that is aligned with a supportive governance structure. The health system infrastructure must also be appropriately resourced to accomplish its objectives, including sufficient staffing, technology, and other resources needed to meet the CDR goals.

CDR work may occur across multiple care settings, including inpatient hospitals, long‐term or skilled nursing facilities, ambulatory primary care clinics, ambulatory surgery centers, or in the community and home (e.g., via pharmacies, home health, and telehealth). An important area to monitor is facility closure and changes to clinical service offerings^31^, ^76^ because maintaining facility and service access may be prioritized as a social objective over reducing costs, improving quality, or increasing efficiency. Because of concerns that consolidation by health systems or private equity firms may lead to the elimination of less profitable service lines, growth in the volume of high‐profit services, and the eventual sale or closure of the institution, regulators and policymakers may need to place conditions on consolidation approvals. There remain concerns that consolidation by health systems or private equity firms may lead to the elimination of less profitable service lines, growth in the volume of high‐profit services, and the eventual sale or closure of the institution.^77^, ^78^

At the core of the Figure are the specific, measurable CDR approaches and interventions that health systems propose and choose to implement to improve outcomes. A recent study found significant heterogeneity in motivation and implementation of these CDR approaches across a purposive sample of 24 health systems with only a small number of these organizations engaged in redesign efforts consistent with the vision of holistic and systematic production function changes, pointing to the importance of the ability to measure and monitor various CDR efforts and evaluate their impact.^8^ This mixed methods study identified 13 CDR intervention categories that could be measured and assessed for purposes of understanding how a health system's production function is changing or to prospectively understand how a system seeking consolidation proposes to change.^8^, ^50^, ^54^, ^57^, ^60^, ^61^, ^62^, ^63^, ^67^, ^68^, ^70^, ^79^ The CDR interventions do not exist in silos but are often complementary. For example, some health systems modify their production goals to pursue a holistic population health strategy, using data and analytics to identify patients who are at high risk for poor outcomes, high users of care, or those with gaps in guideline concordant preventive screening or who can be enrolled in chronic care management or team‐based care programs.^8^ Another intervention that gained considerable traction during the global COVID‐19 pandemic is the use of virtual health care and mobile technology in care encounters. Although telehealth technology has existed for decades, it was never implemented at scale until the COVID‐19 pandemic forced health systems to modify their care production functions, including changes in appointment scheduling, privacy and security, and provider training, to improve access to care.^46^, ^80^, ^81^ Most CDR interventions can be resource‐intensive and are typically not “off‐the‐shelf” solutions.

For example, a common goal of many health system consolidations observed to date includes standardizing the EHRs and IT systems across the different existing and acquired component parts of the system including hospitals, ambulatory physician practices, and other ancillary provider sites. Research evidence has shown that although such standardization makes logical sense, the cost and implementation timeline is often not met and sometimes fails altogether.^82^ For example, Los Angeles based Cedars‐Sinai Medical Center's attempt in 2003 to implement a computerized order entry system was met with significant resistance by hundreds of doctors who claimed the system would actually harm patients and create more inefficiencies in care delivery.^82^, ^83^ The Cedars‐Sinai case example is just one of many in health care where well‐intended process/production improvements can actually result in underachieving the desired quality and efficiency outcomes, possibly leading to increased care costs. Bates described the challenge of implementing effective innovations to an organization's production function when describing the postmortem of the Cedars‐Sinai experience:

Regardless of the size of the practice or practice group making the transition, implementation of an EHR is still difficult, and the consequences of a failed or suboptimal health information technology implementation can be severe. Furthermore, the requirements to achieve a successful transition are often underestimated by the administrative and clinical leadership.’’^82^

When health systems consider multidimensional ways to implement CDR interventions to change their production functions, three different types of approaches are often considered: the “process focused,” “solution focused,” and “problem focused” changes as noted in the Figure.^84^ Process focused approaches try to achieve standardization and efficiency with an existing process. Solution focused approaches require innovative thinking about new ways to provide care, and problem focused approaches address situations in which customers (patients) have unmet needs that are not clearly identified.^84^ The desired end goal should guide interventions and determine a balance among process improvement methods, the development of new services within the existing systems, and the creation of new systems for new stakeholders.

An essential element of the CDR framework is the time dimension because merging or acquired entities may be reluctant to change and/or have very different practice cultures, and health systems often underestimate the time and costs required to enact lasting and meaningful change to their production functions. For example, the desire to systematically implement team‐based care models, including integrating behavioral health specialists, pharmacists, social workers, and other care team members, is a noble goal, but these workers may be in short supply. Without proper workforce assessment, a health system's plan may look good on paper but be impossible to implement. In addition, many health care interventions fail because of inadequate planning for implementation issues such as sustainability (whether the intervention can be sustained after the initial efforts) and fidelity (whether the intervention has been implemented as designed). In addition, intervention dose, adoption, and reach are all important implementation factors that impact long‐term outcomes, as small‐scale efforts are unlikely to tip the scales and meaningfully improve quality at the system level.^85^, ^86^, ^87^ The CDR framework explicitly accounts for key implementation factors and allows for the differing intervention choices by health systems including design quality (how well the intervention is assembled and presented), intensity (the depth of intervention), and engagement (the commitment or seriousness of the intervention recipients).^85^

As with innovative production function changes in any industry, starting with small tests before moving to scale and monitoring ongoing interventions and their progress is usually necessary. The bottom of the Figure illustrates that concurrent data and empirical evidence needs to be monitored by health systems and should be fed back through a learning health system framework to inform practice and decision making and to make “real time” implementation changes as necessary to achieve stated goals.^88^, ^89^ Finally, as noted across the top of the framework, there are a number of factors external to health care systems that facilitate or impede the CDR efforts of health systems including the policy/regulatory context at various levels, which may provide resources and incentives (or disincentives) to implement CDR, such as the inherent economic incentives present in the local market and regional environment. Although many external factors apply similarly across geographies and markets (e.g., Medicare policy), others vary by state and within a specific health care market context.

## From Theory to Implementation: Practical Utility of the CDR Framework for Regulators and Policymakers

The CDR framework provides a conceptual starting point for regulators and policymakers to appreciate the complexity of health system production and the connection of CDR innovations to health systems’ production costs and care quality, including how those should be tied to transaction prices to some degree, even in imperfectly competitive health care markets. We acknowledge, however, that although valuable conceptually for understanding the complexity of health care production, the framework as presented is too detailed and cumbersome to be implemented in practice because it would be impossible for all aspects depicted in the framework to be measured. In the next section, we provide practical suggestions for CDR measurement, including suggestions for how existing data and measurement approaches related to care quality and transaction prices can and should be modified to improve policymaking and regulation. We acknowledge that given both the complexity of the task and the limited current use of existing information and data, what we offer is more of a starting point for catalyzing a discussion than a turn‐key finished product.

## Measuring CDR

To fully understand production and production costs, one ideally needs detailed information about the inputs used in production (e.g., labor, technology, disposable consumables, management and entrepreneurship), the costs of these inputs (e.g., wages for specific types of workers, the cost of capital investments for HIT), and the outputs produced in production. In terms of the feasibility of such data collection, Medicare does currently require all licensed acute care hospitals, home health agencies, and skilled nursing facilities to submit Medicare cost reports with detailed information about hospital revenues; certain types of hospital costs, including wages or capital investment expenditures; and details about how many patients are provided care in the ambulatory and inpatient settings.^90^, ^91^, ^92^ Although this level of granularity for health care systems is feasible in principle and has been used in some studies to understand hospitals’ cost structure at a high level, the information is not by itself detailed enough to make strong conclusions about the overall efficiency of hospital production or its quality of care.^91^ The decision of how granular one should get with data reporting requirements is a key decision point for policy and regulatory discussions.

Production output identification and measurement is also particularly tricky as one must determine the level for which production is being measured and how granular one wants to get in capturing these details. For example, at the health system level, one might measure key population‐level outcome characteristics such as the proportion of a health system's patients who are compliant with recommended preventive care screenings. At the facility level, such as a hospital, one might also look at aggregate level measures such as readmission rates that reflect the proportion of all discharged patients who need to be readmitted to a hospital within a specific time window (e.g., 30 days), a measure believed to be a marker for poor quality of care, as well as discharge planning and postdischarge follow‐up care. One could also look at the procedural level of, for example, a knee replacement surgery, which may be done in a hospital setting or an outpatient ambulatory surgical center. For this procedure, one might look at outcomes beyond surgical volumes including patient experience and postsurgical function and mobility, the need for additional surgical follow‐up and revision, and the amounts paid for the surgical procedure. The point is that there are many decisions to be made, and these decisions should be driven by the overall purpose and feasibility of data collection including implementation costs. By purpose, we mean that if a regulator makes a judgment about whether to allow a proposed health system consolidation, it might be important to require the proposed consolidator to detail the expected change in outcomes and to specifically justify what production process and input changes are planned to lead to these outcome improvements. The regulator might even condition approval of the consolidation on the need to document ex‐post the actual implementation of the proposed process and input changes or demonstrate that production efficiencies are realized and are passed along to payers in the form of lower transaction prices. Because health systems are multiproduct firms with so many practice sites, patient populations, providers, and types of care provided, the challenge is how to practically form health system‐level measures that capture these details. Solving this challenge is beyond the scope of the current paper, but it is important to note that significant research has been published regarding approaches to aggregating multiple individual measures into composite measures for the purpose of formulating valid and reliable latent (i.e., unobserved) constructs reflective of the parent organization.^93^, ^94^, ^95^, ^96^, ^97^ This work, married with existing data and new data collection, could prove useful in helping regulators and policymakers to systematically understand the important constructs of “health system quality” and “health system efficiency.” For the intended purposes of regulators and policymakers, the concept of change is important because it implies both longitudinal measurement and a comparison with a status quo scenario, presumably a time period before an event such as a merger or acquisition, or before a producer implements a CDR intervention. Again, solving for these details is beyond the scope of the paper, but, in order to be useful, data and measures that are produced from the data, including composite measures, will likely need to be dynamic in nature rather than static.

### Capturing CDR Innovations

Table 1 describes 13 CDR activities identified in a prior study of a purposive sample of health care delivery systems in which the focus was on how systems were changing how care is produced and delivered. As described in that study, these 13 categories were formulated from more granular details of changes to the health systems’ production functions as detailed in key informant interviews and secondary document reviews.^8^ These are the same 13 CDR interventions represented in Figure 1, which, as described previously, are typically implemented with the goal of improving existing processes, solving existing problems, or innovating to improve care quality or reduce care costs. The aforementioned study was not dynamic in nature but was instead focused on a health system's strategic plans and actual implementation during a specific timeframe. Still the study was able to assess where each system stood on these 13 dimensions during the particular time window examined, suggesting that the measurement approach could be replicated across multiple time periods and updated, if necessary, for possible new CDR activities. For example, some of the health systems examined had systemic processes to ensure that evidence‐based clinical guidelines were followed, whereas others were more fragmented in their approach to following such guidelines. In terms of the use of data and analytics to guide care management approaches and to assess care quality and outcomes, systems are assigned one of three levels: mature, developing, and immature. Similar assessments and categorizations were used for the other 11 CDR activities identified in the study.

**Table 1:** Definition and Assessment of 13 CDR Activities^a^

CDR Activity	Definition	Assessment Categories
Addressing social determinants of health	Efforts to address ≥1 nonmedical needs of patients	Area of emphasis: HS reports a strong emphasis on efforts targeting patients’ SDH
		Some efforts: HS reports limited efforts targeting patients’ SDH
		Little or no efforts/unknown: HS reports little or no efforts targeting patients’ SDH
Care coordination and management	Efforts to manage/coordinate care across providers and settings	Established: HS reports established care coordination efforts in place
		Developing/limited: HS reports developing care coordination efforts and/or care coordination is in place for limited patient population(s)
Cost and efficiency management	Efforts to manage cost of care (e.g., reducing low value and/or medically unnecessary care, waste, and/or hospital [re]admissions)	Area of emphasis: HS reports that cost reduction is a major focus, or reports that there are well‐established, systemic cost, and efficiency initiatives
		Some efforts: HS reports multiple initiatives around cost and efficiency, sometimes with limited reach, but does not emphasize it as a major area of focus
		Low focus/unknown: HS does not report explicit initiatives focused on cost and efficiency
Data‐driven care and predictive modeling	Use of data and metrics in designing care approaches and/or evaluating care quality and outcomes	Mature: HS reports systemwide use of data/analytics for care design/evaluation
		Developing: HS reports systemic data and analytic capabilities in development, but currently used only in specific areas for care design/evaluation
		Immature: HS reports limited use of data/analytics
Following evidence‐based guidelines	Scientific evidence is used to guide care practices	Systemic use: HS reports systemwide approaches for monitoring and using evidence
		Fragmented use: HS reports monitoring/using evidence in specific areas of care
Identifying and managing the health of populations	Efforts to provide preventive, therapeutic, and diagnostic care for a defined group to improve and maintain the health of the population	Mature: HS reports population health management is a systemic focus and/or reports multiple well‐established population health management initiatives
		Developing: HS reports building population health capabilities and/or reports some population health management initiatives in place, but narrow in scope/not used systemwide
		Immature: HS does not report a formal population health management program
Multidisciplinary care teams	Care that is delivered by organized teams of ≥3 disciplines	Wide reach: HS reports team‐based care is widely implemented
		Limited reach: HS reports team‐based care is in development or implemented on a limited basis
		No emphasis/unknown: HS does not report an emphasis on team‐based care or it is not reported
Patient‐centered care	Efforts to center care on the patient perspective	Established: HS reports pathways in place for patients to influence how HS approaches care delivery
		Developing: HS reports transitioning to a patient‐centric approach, but early in implementation
		No data: Patient‐centric approaches not reported by HS
Reducing harm and making care safer	Efforts to reduce harm or increase patient safety	Area of emphasis: HS reports significant activity around patient safety
		Some efforts: HS reports small‐scale or limited systemic patient safety efforts
		Little or no efforts/unknown: HS has few or no reported patient safety efforts
Specialty care integration	Efforts to integrate specialty care with primary care to improve care/outcomes	Area of emphasis: HS reports a significant emphasis on specialty care redesign
		Some efforts: HS reports limited efforts to redesign specialty care
		Little or no efforts/unknown: HS does not report formal efforts to redesign specialty care
Standardizing care and reducing variance	Efforts to reduce or eliminate variation in care	Yes: HS reports minimizing care variation as an explicit area of emphasis
		No efforts/unknown: HS does not report minimizing care variation as an explicit area of activity
Use of formal quality improvement models	Efforts to improve care; use of care improvement models	Area of emphasis: HS reports QI/care quality as an area of emphasis
		Some efforts: HS reports limited QI/care quality efforts
Using telehealth and mobile technology	Provision of care when patient and provider are at a distance	Yes: HS reports employing telehealth activities
		No efforts/unknown: telehealth activities not reported by HS

CDR, care delivery redesign; HS, hospital; QI, quality improvement; SDH, social determinants of health.

^a^ Adapted from Scanlon and colleagues, 2020.^8^

The 13 activities are focused on critical approaches that have been identified in science and by experts and leaders for generating better care outcomes at the health system level. For example, the list includes efforts to use health technology such as standardized patient health records systems, telehealth consultations, and the use of mobile technology for delivering patient care. It also includes systematic attempts to address health care costs by focusing on standardizing (i.e., reducing variation) care delivery to be consistent with guidelines, systematically reducing low value or unnecessary care, and preventing avoidable hospital emergency department use or preventable hospital readmissions. Importantly, this list includes most of the promised interventions that are often proposed when existing health care entities are seeking regulatory permission to consolidate. The fact that these CDR activities can be measured and tracked over time, even if not at a perfect level of granularity, would be a step in the right direction for better understanding health system production and assessing if changes to production materialize as promised. Importantly, and as described in the next section, the measurement and tracking of these CDR activities can be linked and combined with existing data from other sources, including improvements or refinements in the collection of some of those sources, to create a more robust and comprehensive understanding of the outcomes, quality, value, and costs of care that are being produced and provided. Finally, although our model is focused on CDR in health care delivery systems, these CDR activities are implemented in multiple care delivery settings, including hospitals and ambulatory primary care or multispecialty physician clinics, allowing for similar measurement and assessment at those levels should that be the goal of regulators and policymakers.^32^

## Capitalizing on Existing Data and Mandates: Improving and Enforcing Requirements to Report Transaction Prices, Organizational Costs, and Care Quality

Although possible in theory, obtaining detailed granular data about every health care provider's production function, including details on all inputs employed and the prices of these inputs as well as details about the production technology and the associated outputs generated, may not be feasible for two primary reasons. First, the level of administrative oversight and cost to manage this process is likely not feasible, both for government entities that might require the information but also for provider organizations needing to comply. Second, such nuanced detail is often considered proprietary by health care organizations, and providers and those that represent them would likely resist. Instead, we provide concrete suggestions for how CDR innovations may be measured, as these innovations are often promised when seeking approval for consolidation. Beyond these CDR innovations, there are a variety of other tools available to better understand the value of health care dollars spent and to better approximate, even if imperfectly, how transaction prices relate to care production costs. Tracking the longitudinal evolution of health care organizations, including their various component parts as well as ownership, is quite difficult and will become more difficult with the growth of cross‐market and cross‐state mergers and acquisitions as well as increased ownership by private equity. Although the AHRQ publishes a compendium of US health systems that tracks details including hospitals, group practices, outpatient sites, nursing homes, and home health organizations, there are many other provider organizations that do not meet AHRQ's definition of a health system and fall outside of this tracking effort that should be monitored.^98^

In the next section, we discuss several opportunities for better capitalizing on data already being collected or mandated.

### Enforce 2019 Executive Order on Improving Health Care Price and Quality Transparency

President Trump signed this executive order on June 24, 2019, requiring the Secretary of the Department of Health and Human Services (HHS) to order all US hospitals to publicly post standard price information “including charges and information based on negotiated rates for common or shoppable items and services, in an easy‐to‐understand, consumer‐friendly, and machine‐readable format.”^99^ Subsequently, CMS issued rules requiring both hospitals and health care insurers to disclose and publicly post information about prices (i.e., allowed amounts) for hospital services as well as estimated out‐of‐pocket amounts that patients with specific insurance coverage would be responsible for paying.^100^ The effective date for the hospital requirement was January 1, 2021 with the requirement that each hospital would provide accurate, machine‐readable files with information about the specific billing code (e.g., CPT or DRG) and description of the care provided, the hospital's charge, and—importantly—payer specific allowed amounts for these billed services.^101^ The order also required additional specifications to make sure that billing modifiers, drug distribution units, and other details were included to allow for comparability across institutions and was subsequently updated with changes for the calendar year 2024.^102^ To date, compliance with these mandated reporting requirements has been dismal, with Patient Rights Advocate reporting in November 2024 that only 21.1% of a sample of 2,000 hospitals were in compliance with the order, down from an estimated compliance rate of 34.5% in February of 2024.^103^ The US Office of Inspector General also examined compliance with the order and, using a representative sampling strategy, found that only 46% of the 5,879 US hospitals required to report were in compliance.^104^

Access to the type of transaction price information for all hospitals and all insurers as specified in the Trump order should be a game changer in terms of better understanding market‐based pricing and for reducing health expenditures. Although transaction prices do not necessarily reflect the actual cost of production in imperfect markets, as described previously, the ability to examine the variation in transaction prices within a single market for all providers and for all provider‐insurer negotiated allowed amounts can go a long way to help regulators, policymakers, patients, and consumer advocates to address the high costs of health care. For example, under certain reasonable assumptions, such as the assumption that production costs for the same service should not vary based on a patient's particular insurer or that providers will not stay in business in the long run if they are consistently pricing below the actual costs of production, one can use empirical distributions of transaction prices to at least get a sense of a lower bound on production costs. Although not without flaws, this type of information can support more efficient payment and benefit design, such as the reference‐based pricing approaches implemented by the California Public Employees Retirement System (CalPERS) and others.^105^, ^106^, ^107^

### Expand All‐Payer Claims Databases Beyond the 18 States That Have Them Currently

Only 18 states have legislation that mandates all‐payer claims databases (APCDs). An APCD brings together medical, pharmacy, beneficiary enrollment, and sometimes other data such as dental and vision claims data and includes provider charges and related transaction or “allowed amount” price information in multiple care settings and not just in hospitals. For example, although the Trump administration mandate described previously is largely focused on hospital data, APCDs include information for nonhospital settings as well, an important supplement to the hospital price transparency initiatives by the federal government. If the availability of APCDs was expanded to all states to form a coordinated national data system, it would greatly improve the nation's ability to understand price variation in health care markets. Although transaction prices do not necessarily reflect the costs of production or the efficient cost of production in imperfectly competitive markets, variation, when examined across all competitors offering the same services in a given market area, can provide regulators, policymakers, and purchasers with useful information to at least drive transaction prices toward the cost of production, generating significant potential savings in overall health care expenditures. The lack of widespread adoption of APCDs is largely a political issue with resistance from many providers and insurers around disclosing what these entities believe are proprietary contract information. Although approaches to addressing this political argument are beyond the scope of the paper, regulators and policymakers should be encouraged to push for this type of information, which is necessary given the opaqueness of transparency that currently exists.

### Enhance the Development of Care Episode Payment Approaches

Episode‐based payment models are designed to incentivize efficient use of resources and quality improvement by providers and is a way to move beyond transactional “fee for service” billing in which every element of care is reimbursed separately in a claims‐based reimbursement system.^108^, ^109^ Episodes are formed around care for common conditions in which it is generally known and expected in advance that the episode of care, from beginning to end, will necessitate the use of a number of different office visits, procedures, rehabilitation visits, etc. CMS has identified several episodes of care payment models such as the BPCI, which seeks to set a single price for an episode of care to cover all care necessary in the episode bundle.^110^, ^111^ Indeed, it is often the ability to formulate episodes for specific types of care, for example, common joint replacements such as hips and knees, that have allowed for the implementation of referenced‐based benefit design and new reimbursement models. Using claims and clinical chart data to examine episodes of care can be quite useful for understanding the efficient production frontier because bundles examine the specific types of care that goes into an entire episode. For joint replacements, for example, this would include presurgical workups and diagnostics, including measures of appropriateness for surgery; surgical intervention, including details on whether the procedure was done on an inpatient or outpatient basis, as well as the choice and cost of the implantable joint; and postsurgical care such as rehabilitation visits for physical therapy or pain management. Similar to the transaction price data mentioned previously, the ability to link claims and detailed clinical encounter data across settings and providers allows one to assess variation across providers in care for a specific clinical condition, allowing one the ability to better understand and approximate what should be included in an efficiently produced care episode.

### Coordinate Federal Agency Enforcement Activities Using the HHS Chief Competition Officer

In January of 2024, the Secretary of HHS appointed the nation's first chief competition officer for health care, designed to coordinate across many federal agencies involved in aspects of health care competition and to address specific areas of concern.^112^ These specific areas were outlined in a White House fact sheet issued on December 7, 2023, which listed a number of specific examples in which the federal government should coordinate across existing federal silos of responsibility and address how to improve competition in health care or at least prevent the ill‐consequences of a lack of competition.^113^ For example, the fact sheet focuses on changes to reimbursement for Medicare Advantage plans, concerns about private equity ownership in health care, including “anticompetitive roll‐ups” across organizational entities, often manifested in a series of smaller acquisitions and designed to avoid antitrust enforcement. The fact sheet also mentions support for other previously announced efforts such as the transaction price transparency requirements mentioned earlier. It is unclear whether the Trump administration will keep the chief health care competition position within HHS, but, regardless, progress in addressing many of the ideas mentioned in the fact sheet would enhance the regulatory authority and the ability to promote more efficient production and pricing in health care.

### Reach Consensus on Quality Measures for Regulatory Purposes

The quality measurement industry has grown enormously since the mid‐1990s, resulting in a large number of quality measures now available with many entities involved in producing them, including the National Quality Forum (NQF), the National Committee for Quality Assurance, the Joint Commission, the CMS, and various state‐based or local regional entities conducting quality measurement and public reporting. However, there have been serious concerns with the current practice of measurement and reporting. Not only are the measures used in public reporting a burden for providers, but they can also be unreliable and miss important aspects of care delivery.^114^ In the specific context of provider consolidation, to our knowledge, there has never been a consensus process initiated to discuss which quality measures would be the best to use in assessing the impact and for antitrust regulatory purposes. Importantly, such a consensus process could consider the various types of consolidations proposed, possibly involving hospitals, health plans, provider groups, and health systems, as well as how best measures can reflect these complex organizations with many components. Quality measurement organizations such as the NQF have experience in convening workgroups with experts from different fields to discuss the utility of various quality measures. Such a process can be extended to include key personnel from regulatory authorities, such as the DOJ, the FTC, the HHS chief competition officer, CMS, and relevant state agencies, as well as health economists, health care market experts, and patient advocacy organizations. An advisory group could provide guidance about data sources, measures, and the timeframe for expecting changes in the relevant measures postconsolidation or after specific CDR implementation. Importantly, this would be the first attempt to select measures for this purpose rather than try to fit quality measures designed for other purposes such as improvement and payment.

### Improve the Utility of Required Medicare Cost Reports

For many decades, the Medicare program has required certified institutional providers to annually file cost reports to the Medicare program. Reports are required for hospitals, skilled nursing and long‐term care facilities, home health agencies, health clinics, rural, rural health providers, and some others.^35^ The cost reports are designed to help Medicare set reimbursement rates that reflect actual production costs of Medicare providers. These reports require aggregate information about the facility including total revenues, operating expenses, capital expenses, operating margin, number of patients provided care, and the distribution of payers including self‐pay and charity care/bad debt. They also include utilization information such as hospital discharge volume and information on aggregate expenditures, including some information broken down by specific departments.^115^ Although the cost reports are used by the Medicare Payment Advisory Committee and others, they have been criticized for being too aggregate in nature and not as useful for understanding the complexity of multiproduct firms, with studies having found the data contained in the reports to be unreliable, difficult to use when comparing across providers, and in some cases creating an inaccurate picture of hospital finances.^91^, ^116^ The growth of health care systems and the interrelationship among provider organizations at different levels of service, such as hospitals, physician groups, and ambulatory surgical centers, challenges the ability to fully understand production cost details at the service line level and for common episodes of care. Analyses have documented the challenges of using existing reports for these purposes and have made recommendations for how this reporting requirement could be improved to better assist policy and regulation.^117^

### Cost of Enforcement and Legal Authority

While it is possible to conceive of approaches for better monitoring changes in health care transaction prices, quality of care, and care production, it is important to understand the legal authority to do so in addition to understanding the monitoring costs. It seems clear that additional monitoring and enforcement will come with increased costs that must be accounted for and absorbed. Because the goal is to reduce overall health care costs for consumers and payers rather than to raise prices, it will be important to appropriately monetize and account for additional regulatory costs and to appropriate the necessary funding to those charged with such monitoring and enforcement. It should be noted that the costs of regulation and enforcement are routinely accounted for and appropriated in other industries, such as the Securities and Exchange Commission or the Food and Drug Administration, to provide just two of many examples. The cost benefit equation for justifying enhanced monitoring should be clear from a societal perspective. To think about this retrospectively, if such monitoring was in place and reduced the significant trend in health care cost increases, then any amount spent that is less than the savings generated could be considered a beneficial investment. This idea can apply to specific payers as well. For example, if Congress was to appropriate additional resources to the FTC and DOJ to engage in enhanced regulation, that could generate a positive return on investment if the Medicare program reaps the benefit of paying for more efficiently priced health care and a significant reduction in the trend of health care costs for Medicare and Medicaid beneficiaries.

## Limitations

There are important limitations to the proposed CDR framework and other suggestions we make. First, although not all types of CDR activities may be described in the proposed framework, those included come from a recent study and could be supplemented over time based on additional information. Second, implementing the suggested changes would take political consensus to agree on their necessity and provide the necessary congressional actions and resource appropriation to allow for implementation. Third, and most importantly, our proposed framework and suggested policy and regulatory changes are meant to encourage a more robust discussion of feasible alternatives that might help the United States to more meaningfully address the ongoing problem of the high costs of health care, helping to move closer to a time when all Americans can benefit from an improved health care system that provides equity in access to high quality and efficiently produced and priced health care services and health insurance.

## Funding/Support

This work was supported through the RAND Center of Excellence on Health System Performance, which is funded through a cooperative agreement (1U19HS024067‐01) between the RAND Corporation and the Agency for Healthcare Research and Quality. The content and opinions expressed in this publication are solely the responsibility of the authors and do not reflect the official position of the Agency or the US Department of Health and Human Services.
